# *In-vitro* effects of different hyaluronic acids on periodontal biofilm-immune cell interaction

**DOI:** 10.3389/fcimb.2024.1414861

**Published:** 2024-06-13

**Authors:** Xilei Zhu, Anton Sculean, Sigrun Eick

**Affiliations:** ^1^ Department of Periodontology, School of Dental Medicine, University of Bern, Bern, Switzerland; ^2^ Graduate School for Cellular and Biomedical Sciences, University of Bern, Bern, Switzerland

**Keywords:** hyaluronic acid, periodontitis, periodontal therapy, macrophages, periodontal ligament fibroblasts, anti-biofilm activity, anti-inflammation, antioxidation

## Abstract

**Introduction:**

Recent studies have demonstrated a positive role of hyaluronic acid (HA) on periodontal clinical outcomes. This *in-vitro* study aimed to investigate the impact of four different HAs on interactions between periodontal biofilm and immune cells.

**Methods:**

The four HAs included: high-molecular-weight HA (HHA, non-cross-linked), low-molecular-weight HA (LHA), oligomers HA (OHA), and cross-linked high-molecular-weight HA (CHA). Serial experiments were conducted to verify the influence of HAs on: (i) 12-species periodontal biofilm (formation and pre-existing); (ii) expression of inflammatory cytokines and HA receptors in monocytic (MONO-MAC-6) cells and periodontal ligament fibroblasts (PDLF) with or without exposure to periodontal biofilms; (iii) generation of reactive oxygen species (ROS) in MONO-MAC-6 cells and PDLF with presence of biofilm and HA.

**Results:**

The results indicated that HHA and CHA reduced the bacterial counts in a newly formed (4-h) biofilm and in a pre-existing five-day-old biofilm. Without biofilm challenge, OHA triggered inflammatory reaction by increasing IL-1β and IL-10 levels in MONO-MAC cells and IL-8 in PDLF in a time-dependent manner, whereas CHA suppressed this response by inhibiting the expression of IL-10 in MONO-MAC cells and IL-8 in PDLF. Under biofilm challenge, HA decreased the expression of IL-1β (most decreasing HHA) and increased IL-10 levels in MONO-MAC-6 cells in a molecular weight dependent manner (most increasing CHA). The interaction between HA and both cells may occur via ICAM-1 receptor. Biofilm stimulus increased ROS levels in MONO-MAC-6 cells and PDLF, but only HHA slightly suppressed the high generation of ROS induced by biofilm stimulation in both cells.

**Conclusion:**

Overall, these results indicate that OHA induces inflammation, while HHA and CHA exhibit anti-biofilm, primarily anti-inflammatory, and antioxidant properties in the periodontal environment.

## 1 Introduction

Hyaluronic acid (HA), also known as hyaluronan, is a natural polysaccharide molecule, first isolated by Karl Meyer and John Palmer in 1934, and it is the only type of glycosaminoglycans (GAGs) that is not sulphated (Meyer and Palmer, 1934). HA consists of repeating disaccharide units of N-acetyl-glucosamine and glucuronic acid. Depending on the number of composed units, it has a wide range of molecular weight (MW), from a few dimers to millions of Daltons (Da) (Fraser et al., 1997). HA can be divided into high-MW HA (>1000 kDa) and low-MW HA (<500 kDa). Low-MW HA can further fragment into shorter oligomers (Jiang et al., 2011). Furthermore, HA exists in many formulations based on chemical modification of interunit chemical groups, including cross-linked and non-cross-linked (Choi et al., 2015).

HA occurs in many parts of the human body, including the skin, skeleton, joints (Fraser et al., 1997), and oral tissues (Martins et al., 2003). It is one of the primary components of the extracellular matrix (ECM) that regulates normal tissue integrity and development. Through interaction with cell surface receptors, HA plays important roles in inflammation and regeneration (Garantziotis and Savani, 2019). Some well-known HA receptors include homing cell adhesion molecule CD44, toll-like receptors 2/4 (TLR2/4), receptor for hyaluronan-mediated motility (RHAMM), and intercellular adhesion molecule-1 (ICAM-1), involved in cell growth, adhesion, motility together with their downstream signaling pathways (Jiang et al., 2011). HA has been suggested to have diverse biological functions depending on its MW and composition. For instance, HA fragments are believed to be pro-inflammatory and promote angiogenesis in many diseases, whereas high molecular weight HA (HHA) is considered to be more anti-oxidant, anti-inflammatory and anti-angiogenic (Litwiniuk et al., 2016). Due to these properties, HA is now widely used in tissue regeneration as well as in anti-aging and anti-inflammatory agents (Vasvani et al., 2020). In dentistry, HA has been reported to have a beneficial effect on clinical outcomes as an adjunct to non-surgical and surgical periodontal treatment (Bertl et al., 2015; Eliezer et al., 2019).

Periodontitis is characterized as a microbial-associated, host-mediated inflammation leading to periodontal attachment loss, tissue destruction, and ultimately tooth loss (Kinane et al., 2017). Keystone pathogens such as *Porphyromonas gingivalis* (Hajishengallis et al., 2012) along with accessory pathogens initially over-activate the inflammatory response and cause periodontal tissue destruction (Hajishengallis, 2014). However, the microbiome and host inflammatory response, involving networks of cytokines, chemokines and growth factors, are in bidirectional imbalance during disease propagation, and their interaction determines disease regression (Curtis et al., 2020). One of the hallmarks of periodontitis is the complex cell infiltration including polymorphonuclear neutrophils (PMNs), granulocytes, monocytes, and lymphocyte infiltration (Kinane et al., 2017). Macrophages contribute significantly to tissue homeostasis and defense by polarizing into M1 and M2 phenotypes. M1-type cells are activated by interferon gamma (IFN-γ) and lipopolysaccharides (LPS) and secrete pro-inflammatory cytokines such as interleukin (IL)-1β, IL-6; whereas M2-type cells respond to IL-4 and IL-13 and participate in resolution of inflammation as evidenced by high levels of IL-10 and transforming growth factor-β (TGF-β) (Sun et al., 2021). Periodontal ligament fibroblasts (PDLF) are the most abundant cells in the periodontal ligament, which anchors teeth to alveolar bone for support and protection (Beertsen et al., 1997). Aside from its crucial role in periodontal tissue remodeling and homeostasis, PDLF also performs an immunomodulatory role during periodontitis progression by generating immune mediators such as chemokine IL-8. PDLF overexpression of proinflammatory cytokines may lead to amplification of the local inflammation by constantly triggering the immune response (El-Awady et al., 2010).

Clinical studies have demonstrated that the adjunctive application of high molecular weight (MW) HA after instrumentation has favorable effects on clinical outcomes and prevents the recolonization of periodontal pathogens (Eick et al., 2013). *In vitro* findings also revealed that high-MW HA (both cross-linked and non-cross-linked) enhanced the surface roughness of dentine discs with a high survival rate and spreading of PDLF (Mueller et al., 2017). Cells involved in periodontal tissue regeneration, such as PDLF, palatal and gingival fibroblasts, showed high cell viability and boosted proliferation when exposed to high-MW HA, suggesting high biocompatibility of HA for periodontal use (Fujioka-Kobayashi et al., 2017; Asparuhova et al., 2019).

However, considering the diverse functions associated with different molecular weights (MW) of HA, the specific impact of varying MWs of HA on the periodontal environment, as well as its potential mechanisms, remains unclear. The aim of this *in-vitro* study was to investigate the potential effects of different MWs of HA, as well as different formulations (cross-linked vs. non-cross-linked), on interactions between periodontal biofilm and immune cells.

## 2 Methods

### 2.1 HA preparation

Four different HA were used in this study: three non-cross-linked HAs (Bloomage Biotech, Jinan, China) of varying MW: 6 kDa HA oligomers (OHA), low-MW 400 kDa HA (LHA), high-MW 1000 kDa HA (HHA) and one cross-linked high-MW 1000 kDa HA (CHA, Regedent AG, Zurich, Switzerland) containing 18 mg/ml HA.

A concentration of 4 mg/ml HA was used in all cell experiments. In biofilm experiments, for the 4-h biofilm formation, concentrations of 2 and 8 mg/ml HA were used, and in the case of 5-day pre-cultured biofilm, 18 mg/ml HA were applied.

### 2.2 Microorganisms and cultivation

Twelve bacterial species of bacteria were included in the biofilm experiments:

1. *Streptococcus gordonii* ATCC 10558
2. *Actinomyces naeslundii* ATCC 12104
3. *Fusobacterium nucleatum* ATCC 25586
4. *Campylobacter rectus* ATCC 33238
5. *Parvimonas micra* ATCC 33270
6. *Eikenella corrodens* ATCC 23834
7. *Prevotella intermedia* ATCC 2561
8. *Capnocytophaga gingivalis* ATCC 33624
9. *Porphyromonas gingivalis* ATCC 33277
10. *Tannerella forsythia* ATCC 43037
11. *Filifactor alocis* ATCC 33099
12. *Treponema denticola* ATCC 35405

Except for *T. denticola* [cultured in mycoplasma broth (BD, Franklin Lake, NJ)], the other 11 strains were maintained on Schaedler agar plates (Oxoid, Basingstoke, UK) supplemented with 5% sheep blood, in the case of *T. forsythia* additionally with 10 mg/l N‐acetylmuramic acid. All strains were cultured at 37°C in the respective atmosphere: *S. gordonii* and *A. naeslundii* with 10% CO_2_, and the others in an anaerobic incubator.

### 2.3 Activity on periodontal bacteria and biofilm

#### 2.3.1 Determination of minimum inhibitory concentration

The MIC values of different HAs were determined against the above-mentioned bacterial species except for *T. denticola* ATCC 35405. Two-fold dilution series ranging from 0 to 20 mg/ml were prepared for different HAs. The microorganisms were then suspended in a two-fold concentrated Wilkins-Chalgren broth (Oxoid) and combined with varying concentrations of the different HAs at a 1:1 ratio. After incubating anaerobes for 24 h and aerobes for 18 h, the MIC values were determined as the lowest concentration with visible growth inhibition.

#### 2.3.2 Biofilm assays

Two aspects of the 12-species periodontal biofilm were explored: (1) influence on biofilm formation and (2) influence on pre-existing biofilm.

(1) Influence on biofilm formation: wells of a 48-well plate were covered with 25 μl/well 1.5% bovine serum albumin (BSA)/0.67% mucin solution at room temperature for 1 h to generate a proteinaceous surface layer. Then, 50 μl/well of the different HA solutions (2 mg/ml and 8 mg/ml) was used), were added for another 30 min incubation. Thereafter, microbial suspension mixed with nutrient broth (Wilkins-Chalgren broth) in a volume ratio of 1:9 was added, 450 μl per well, meaning the final concentration of HA were 0.2 and 0.8 mg/ml respectively. Subsequently, the plate was incubated anaerobically at 37°C. The microbial suspension consisted of one part *S. gordonii*, two parts *A. naeslundii*, eight parts *T. denticola*, and four parts the other nine species, all of which were suspended in 0.9% NaCl at McFarland 4. After 4 h, the biofilms were scraped from the well surface after careful washing and resuspended in 0.9% NaCl. The biofilm suspension was serially diluted and sub-cultured on agar plates for colony forming units (CFU) assessment after one week of anaerobic incubation.
(2) Influence on pre-existing biofilm: wells of the 48-well plate were coated with protein solution as described above, and 450 μl/well of microbial suspension mixed with Wilkins–Chalgren broth (volume: 1:9, as stated above) was added. The plates were then cultured in an anaerobic incubator for 5 days, at day 3, the medium was replaced by fresh one supplemented with *P. gingivalis*, *T. forsythia*, and *T. denticola*. At day 5, the nutrient broth was carefully removed, and the biofilm was gently washed. Then, 50 μl//well HAs at 18 mg/ml (according to the concentration of CHA) was added to the biofilm for 1 min, followed by 450 μl/well Wilkins-Chalgren broth. After 1 h of anaerobic incubation, the biofilms were scraped from the well surface and resuspended in 0.9% NaCl into aliquots: one part for CFU, one part for biofilm mass quantification via crystal violet staining (Merritt et al., 2005), and one part for biofilm metabolic activity via Alamar blue staining as previously described (Pettit et al., 2005).

#### 2.3.3 Live/dead staining

Based on the significant results of biofilm formation in the HHA and CHA groups, the 4-h biofilms treated with HHA and CHA (8 mg/ml) were stained using LIVE/DEAD^®^ BacLight Bacterial Viability Kits (Molecular Probes, Life technologies, USA) according to the manufacturer’s instructions. The images were then taken using a Zeiss LSM 710 confocal microscope (Carl Zeiss) with oil immersion in two fluorescence channels (green and red). Green staining indicates live cells while red staining indicates dead cells. *Imarisviewer* software (Bitplane, *IMARIS* 10.0.0) was used for additional visualization.

### 2.4 Cell culture

The human monocytic cell line MONO-MAC-6 obtained from DMSZ (Braunschweig, Germany) was cultivated in RPMI 1640 medium supplemented with 10% double-heat-inactivated fetal bovine serum (FBS), 1 mM non-essential amino acids, 1 mM sodium pyruvate and 10 μg/ml human insulin (Invitrogen; Carlsbad, CA, USA).

Human PDLF were isolated from freshly extracted and donated premolar teeth from systemically healthy young adults undergoing orthodontic therapy. The individuals had been informed about the use of teeth for research purposes and completed a written consent form. According to the criteria of the Cantonal Ethical Committee (KEK), there is no need for additional approval if the biomaterials are classified as “irreversibly anonymized”. The primary cell culture procedure was applied as previously described (Lin et al., 2020). Briefly, tissues from the mid-third of the teeth were washed three times with PBS before being minced into 1-mm^3^ cubes. The cubes were then grown on average in Dulbecco’s modified Eagle’s medium (DMEM) (Invitrogen; Carlsbad, CA, USA) supplemented with 10% FBS supplemented with an antibiotic-antimycotic solution (Gibco, Thermo Fischer, MA, USA) containing 100 units/ml penicillin, 100 μg/ml streptomycin, and 0.25 μg/ml of amphotericin B as the final concentration. Cells from at least three donors were maintained.

Before being exposed to test substances, all cell strains were grown at 37°C with 5% CO_2_ and starved overnight in 0.5% FBS/DMEM or 0.5% FBS/RPMI 1640 being also the “regular medium” in the experiments.

#### 2.4.1 MTT assay

The cell viability was assessed using the MTT assay (Mosmann, 1983). PDLF were seeded in 96-well-plates at a density of 1x10^5^ cells per well and allowed to reach confluency for at least 24 hours. On the second day, the cells were gently washed twice with PBS, followed by a change to 100 μl/well HAs-medium (different HAs with a concentration ranging from 0 to 4 mg/ml in 0.5% FBS-DMEM). In the case of MONO-MAC-6 cells the cells were resuspended in HAs-medium (HAs in 0.5% FBS-RPMI). After 4 h treatment in HAs-medium, 10 μl/well MTT solution (final concentration, 0.5 mg/ml) was added for additional 2 h-incubation. The dark blue formazan crystals formed within viable cells were solubilized using lysis buffer (20 μl of 3% SDS and 100 of acid-propanol) and well mixed before measuring absorbance at 570 nm relative to the reference wavelength of 630 nm with a microplate reader (Agilent, CA, USA). The data was presented as a percentage (%) of control (untreated cells).

#### 2.4.2 Release of inflammatory cytokines and HA receptors in MONO-MAC-6 cells and PDLF

In general, MONO-MAC-6 cells and PDLF were divided into two groups: (a) biofilm stimulated group (BS group): cultivated with different HAs-media under biofilm stimulations; (b) non-stimulated group (NS group): only HAs-medium. Quantitative PCR (qPCR) and ELISA were used to assess the inflammatory cytokine expression levels between the BS and NS groups, while qPCR was used to detect HA receptors.

First, a 48-h periodontal biofilm was created as previously indicated. For MONO-MAC-6 cells in BS groups, cells in HAs-medium (4 mg/ml different HAs in regular medium) were seeded into the wells with the formed biofilms after removing the microbial nutrient medium and properly washing. The cells in the NS group were seeded directly into the wells and cultured under the same conditions. For PDLF, the 48-h biofilms were harvested, adjusted to McFarland 4 and exposed to ultrasonication for 20 min and then centrifuged at 4000 g for 5 min to extract the biofilm supernatant. PDLF were seeded into wells to generate a monolayer for 24 h. On the second day, the medium was exchanged to HAs-medium (4 mg/ml), with or without 10% biofilm supernatant.

For ELISA, the cell suspensions were collected after certain incubation times (2 h, 4 h) and centrifuged at 8000 g before supernatants were obtained and stored at -80°C. The IL-1β and IL-10 protein levels were measured in the MONO-MAC-6 cells supernatants, and IL-8 in the PDLF supernatants using ELISA kits (R&D Systems Europe Ltd., Abingdon, UK) as instructed. For the mRNA expression, total RNA was extracted from both cells after 2 h of incubation using the innuPREP RNA Mini Kit 2.0 (Analytic Jena GmbH, Germany). The GoScript™ Reverse Transcription System (Promega, Madison, WI, USA) was then used to reverse cDNA from 1000 ng RNA. GoTaq^®^ qPCR Master Mix (Promega) was used along with the QuantStudio 3 RT-PCR System (Thermo Fischer, Waltham, MA, USA) to perform qPCR. GAPDH was used to normalize gene expression, and the 2^-△△CT^ method was used to assess the relative expression of the respective gene (Livak and Schmittgen, 2001). The IL1B and IL10 mRNA expression levels were measured in MONO-MAC-6 cells, and IL8 in PDLF; the HA receptor genes (CD44, RHAMM, TLR2, TLR4, and ICAM1) expressions were determined in both cells; the primers are indicated in **Table 1**.

**Table 1:** Primer sequences used for qPCR.

Gene	Forward/Reverse primers	Primer sequences 5’-3’	References
*IL8*	F	GAG AGT GAT TGA GAG GTG GAC CAC	(Parisi et al., 2022)
	R	CAC AAC CCT CTG CAC CCA GTT T	
*IL1B* *IL10* *TLR2* *TLR4* *ICAM1*	F R F R F R F R F R	TAC GAA TCT CCG ACC ACC ACT ACA G TGG AGG TGG AGA GCT TTC AGT TCA TAT G GCC TAA CAT GCT TCG AGA TC CTC ATG GCT TTG TAG ATG CC GGG TCA TCA TCA GCC TCT CC AGG TCA CTG TTG CTA ATG TAG GTG CAG AGT TGC TTT CAA TGG CAT C AGA CTG TAA TCA AGA ACC TGG AGG AGC GGC TGA CGT GTG CAG TAA T TCT GAG ACC TCT GGC TTC GTC A	(Fernandez-Botran and Vetvicka, 2000) (Chhabra et al., 2008) (Sabroe et al., 2002) (Sabroe et al., 2002) (Ma et al., 2021)
*CD44*	F	GAC CTC TGC AAG GCT TTC AAT A	# M59040.1
	R	CAA AGG CAT TGG GCA GGT CT	
*RHAMM*	F	AGG ACC AGT ATC CTT TCA GAA ATC	# BC017793.1
	R	AGT GCA GCA TTT AGC CTT GC	
*GAPDH*	F	GAC AGT CAG CCG CAT CTT CT	(Shen et al., 2010)
	R	TTA AAA GCA GCC CTG GTG AC	

#### 2.4.3 Reactive oxygen species assay

The MONO-MAC-6 cells or PDLF were incubated for 1 h in HAs-medium (4 mg/ml) or regular medium with or without biofilm stimulation before total ROS generation was quantified. The ROS assay was carried out using the Cellular ROS assay kit (Red Fluorescence) (Abcam, Cambridge, UK) following the manufacture’s instruction with a Varioskan™ LUX multimode microplate reader (Thermo Fisher Scientific, USA). The wavelengths of excitation and emission were 520 nm and 605 nm, respectively.

### 2.5 Statistical analysis

All experiments were repeated in at least two independent experiments with each quadruplicate. The data was displayed as mean and standard deviation (SD), and a log10 transformation was performed for CFU analysis.

The graphs were created with GraphPad Prism 9 (Graphpad Software, Bosten, MA, USA). SPSS 28.0 (IBM, Chicago, IL, USA) was used for statistical analysis. Following a Shapiro-Wilk test for normality, the one-way analysis of variance (one-way ANOVA) with *post-hoc* Tukey was conducted. The focus was on comparing the respective HA group with the control group and comparing the CHA with the HHA group. A *p*-value below 0.05 was considered statistically significant.

## 3 Results

### 3.1 Periodontal bacteria and biofilm

According to the MIC test, a concentration up to 10 mg/mL did not impede visible bacterial growth of all tested bacterial species.

Two aspects of periodontal biofilm were investigated in terms of the potential effects of different MW as well as of the cross-linked structure of HA: biofilm formation and biofilm destruction.

#### 3.1.1 Early biofilm formation

To assess the impact on biofilm formation, surfaces were coated with HA solutions (each 2 mg/ml and 8 mg/ml) prior adding the microbial suspension for biofilm formation.

**Figure 1:**
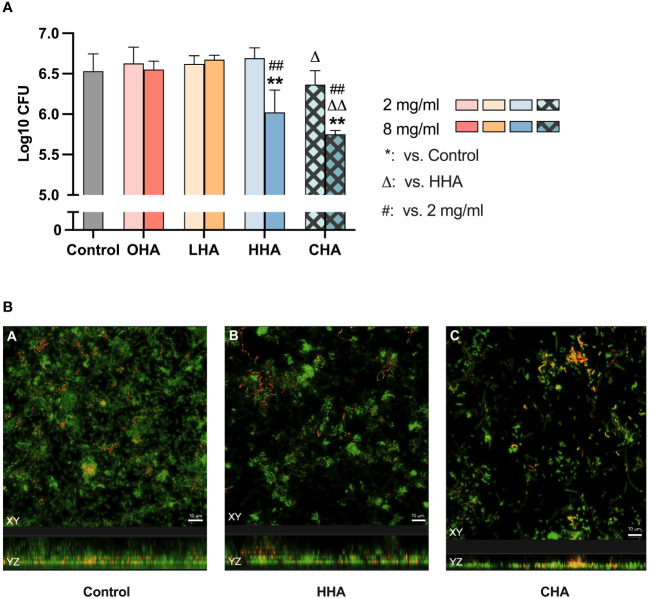
Impact of coating with 2 mg/ml and 8 mg/ml hyaluronic acid (HA) (OHA: 6 kDa, non-cross-linked; LHA: 400 kDa, non-cross-linked; HHA: 1000 kDa, non-cross-linked; CHA: 1000 kDa, cross-linked) on early (4 h) periodontal biofilm formation: **(A)** colony forming units (CFU; mean and SD). ** *p*<0.01 vs. control group, △/△△ *p*<0.05/0.01 vs. respective concentration HHA. **(B)** Confocal laser scanning microscopy images using live/dead staining assay (green live cells, red dead cells). **(A)** Control biofilm (ii) Biofilm treated with 8 mg/ml HHA. (iii) Biofilm treated with 8 mg/ml CHA. Scale bar = 10 μm.

As demonstrated in **Figure 1A**, only the 8 mg/mL HHA and CHA solution resulted in a significant reduction (-0.51 log10 by HHA and -0.78 log10 by CHA) of CFU compared to the control (both *p<*0.001). Comparing HHA and CHA, lower CFU counts were always found after the coating of 2 and 8 mg/ml CHA vs. the respective concentration of HHA (by -0.33 log10, *p*=0.009 and by -0.27 log10, *p*=0.028).

Considering the reducing effect of 8 mg/ml HHA and CHA, live/dead staining was performed on 4 h-biofilms where the surfaces were coated with 8 mg/ml of HHA and CHA. The confocal images in **Figure 1B** indicated that both HHA and CHA groups resulted in lower density and thickness of the 4-h biofilms compared to the control group, with CHA having the lowest values.

#### 3.1.2 Mature biofilm destruction

To evaluate the effect of HA on mature biofilm, 18 mg/ml HAs, simulating the concentration of the commercial product was applied to the pre-existing five-day biofilm for 1 min.

**Figure 2:**
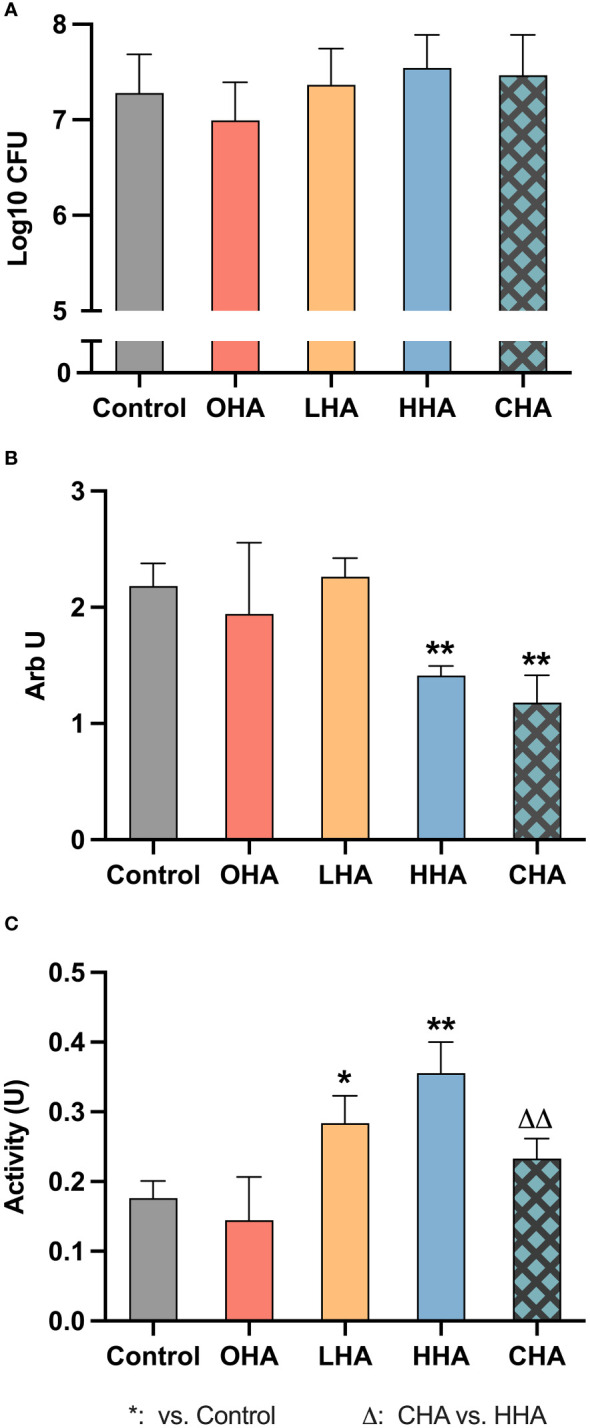
Impact of 18 mg/ml for 1 min hyaluronic acid (HA) (OHA: 6 kDa, non-cross-linked; LHA: 400 kDa, non-cross-linked; HHA: 1000 kDa, non-cross-linked; CHA: 1000 kDa, cross-linked) on the destruction of pre-formed five-day periodontal biofilm: **(A)** Colony forming units (CFU) counts, **(B)** quantity, and **(C)** metabolic activity The CFU values were subjected to a log10 transformation. The results are presented as Mean ± SD, */** *p*<0.05/*p*<0.01 vs. control group, △△ *p*<0.01 vs. HHA.

As shown in **Figure 2A**, HAs did not significantly influence the CFU counts in the 5 d-biofilm. Regarding biofilm mass, only HHA and CHA induced a significant reduction (each *p<*0.001, **Figure 2B**). CHA seemed to reduce more biofilm mass than HHA, but there was no statistical significance. The metabolic activity of the biofilm increased in LHA and HHA group (each *p<*0.001, **Figure 2C**). The metabolic activity was less in CHA than in HHA group (*p*<0.001), **Figure 2C**.

### 3.2 Immune interaction between MONO-MAC-6 cells and biofilm stimulation

The effect of different HAs on the inflammatory response of a monocytic cell line (MONO-MAC-6) with or without a periodontal biofilm challenge was investigated in four aspects: relative expression of inflammatory cytokines, expression of HA receptors, and oxidative stress.

To confirm sufficient cell viability in the experimental conditions, the MTT test was performed on MONO-MAC-6 cells being exposed to 4 mg/ml HA for 4 h. The viability of MONO-MAC-6 cells did not remarkably decrease, it remained more than 75% throughout (data not shown in detail).

#### 3.2.1 Expression of IL-1β and IL-10 in MONO-MAC-6 cells

To assess inflammatory cytokine expression, we investigated the expression of both IL-1β and IL-10 at the protein and mRNA levels in MONO-MAC-6 cells. MONO-MAC-6 cells in HAs-medium (4 mg/ml) or without HA (control group) were exposed to periodontal biofilm (BS) or not (NS) for 2 h and 4 h for protein detection and 2 h for mRNA detection.

##### 3.2.1.1 Protein expression

**Figure 3:**
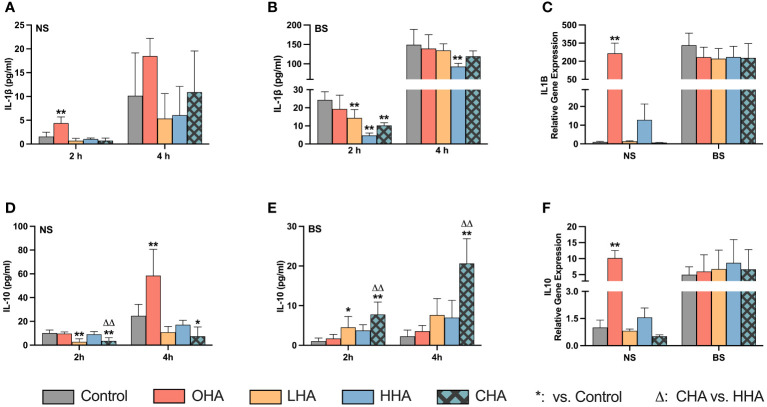
Effect of 4 mg/ml hyaluronic acid (HA) (OHA: 6 kDa, non-cross-linked; LHA: 400 kDa, non-cross-linked; HHA: 1000 kDa, non-cross-linked; CHA: 1000 kDa, cross-linked) on protein **(A, B, D, E)** and mRNA **(C, F)** expression of interleukin-1β [IL-1β **(A, B)**, ILB **(C)**] and interleukin-10 [IL-10 **(D, E)**, IL10 **(F)**] in a monocytic cell line (MONO-MAC-6 cells) with **(B, C, E, F)** or without **(A, D, E, F)** periodontal biofilm after 2 h and 4 h (only protein levels) of stimulation. NS, non-biofilm-stimulated; BS, biofilm-stimulated. Mean ± SD, */** *p*<0.05/*p*<0.01 vs. control, △△ *p*<0.01 vs. HHA group.

At the protein level, in the absence of HA, MONO-MAC-6 cells released more IL-1β (**Figures 3A, B**, both *p<*0.001 at 2 h and 4 h) and less IL-10 (**Figures 3D, E**, *p<*0.001 at both 2 h and 4 h) when challenged with periodontal biofilm for 2 h and 4 h, compared to those without biofilm.

In the NS group (**Figures 3A, D**), higher IL-1β and IL-10 levels were observed over time in the OHA group, with a significant difference noted at 2 h for IL-1β (*p<*0.001), and at 4 h for IL-10 (*p<*0.001). However, LHA slightly decreased the amount of IL-10 at both 2 and 4 h compared to the control (*p<*0.001 at 2 h). No significant changes were observed in MONO-MAC-6 release of IL-1β and IL-10 for the HHA group; the CHA group decreased the IL-10 levels at 2 h (*p*<0.001 vs. control, and *p*<0.001 vs. HHA) and at 4 h (*p*=0.044 vs. control).

In the BS group (**Figure 3B**), at 2 h, the level of IL-1β decreased in an MW-dependent manner with HHA having the lowest expression (*p=*0.001 for LHA, HHA and CHA groups vs. BS control). A similar trend was observed among the HA groups after 4 h, but only HHA reached statistical significance (*p=*0.001). Regarding IL-10 levels in the BS group (**Figure 3E**), HA increased the IL-10 expression in a MW-dependent behavior, with CHA had the highest increasing effect at both 2 h and 4 h, *p*<0.001 vs. control and *p*=0.003 vs. HHA.

##### 3.2.1.2 mRNA expression

At the mRNA level (**Figures 3C, F**) indicated that biofilm stimulation primarily upregulated the gene expression of IL1B (333-fold change for IL1B, *p=*0.029) and by trend of IL10 (5.0-fold change, *p=*0.108). In the presence of HA, OHA significantly elevated the mRNA expression of both IL1B and IL-10 in NS groups by 266-fold change and 10.2-fold change respectively (both *p<*0.001 vs. NS control). In BS groups, HA did neither influence the IL1B expression nor the IL10 mRNA levels.

#### 3.2.2 Expression of HA receptors in MONO-MAC-6 cells

**Figure 4:**
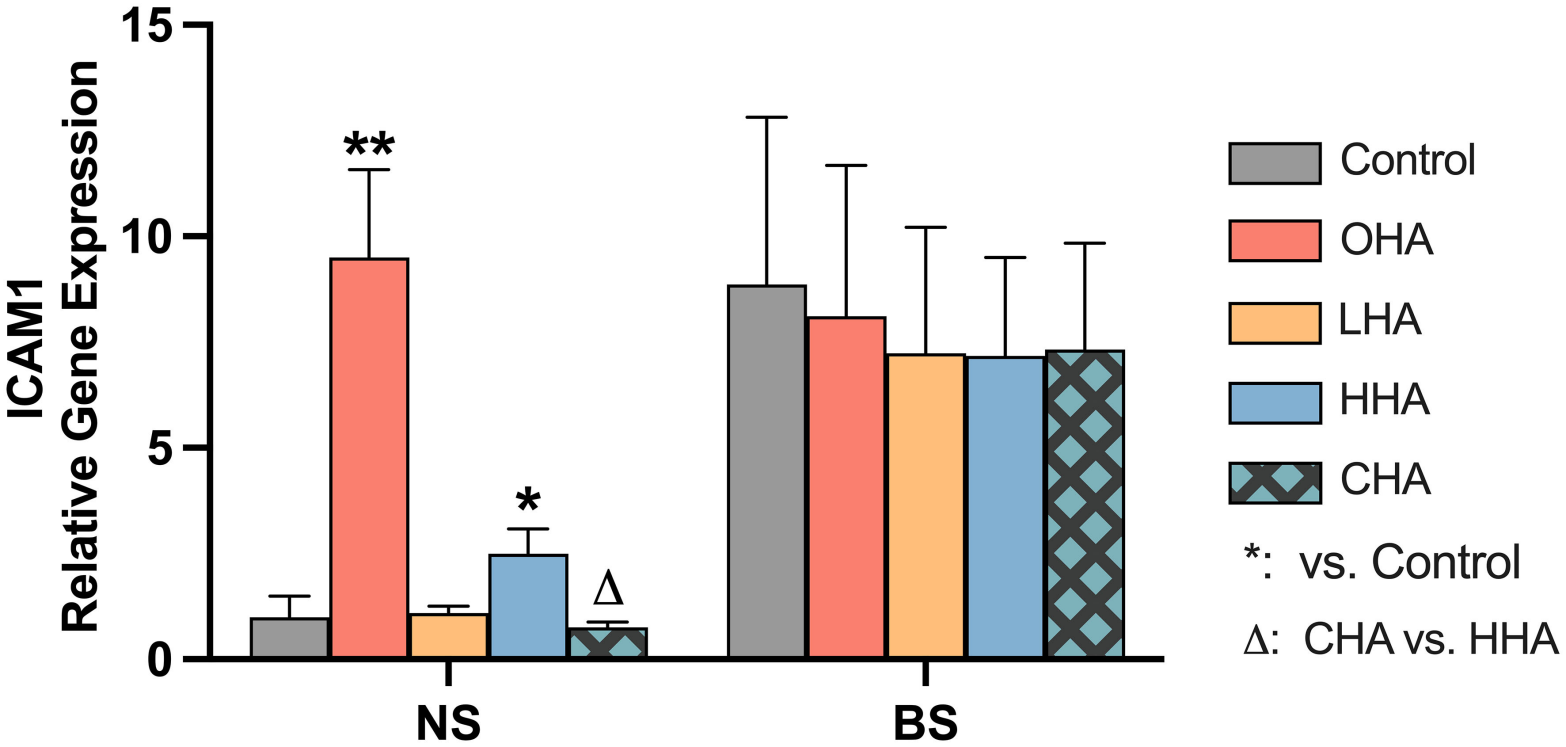
Effect of 4 mg/ml hyaluronic acid (HA) (OHA: 6 kDa, non-cross-linked; LHA: 400 kDa, non-cross-linked; HHA: 1000 kDa, non-cross-linked; CHA: 1000 kDa, cross-linked) on the mRNA expression levels of a HA receptor gene – ICAM1 in a monocytic cell line (MONO-MAC-6 cells) with or without biofilm after 2 h stimulation. NS, non-biofilm stimulated; BS, biofilm-stimulated. Mean ± SD, */** *p*<0.05/*p*<0.01 vs. control, △ *p*<0.05 vs. HHA group.

The mRNA expression of five major HA receptor genes (CD44, RHAMM, TLR2, TLR4, and ICAM1) was investigated in MONO-MAC-6 cells. The cells were stimulated with or without biofilm and were treated with or without HA. Different HAs (4 mg/ml) for each mRNA expression assay were used, with an incubation time of 2 h. However, among the studied receptors, only ICAM1 was affected by the stimuli (**Figure 4**), the expression of the other four receptor genes (CD44, RHAMM, TLR2, and TLR4) were not influenced by either HA or biofilm stimulation (data not shown).

Biofilm stimulation increased ICAM1 mRNA expression in MONO-MAC-6 cells by 8.9-fold (*p=*0.028) without supplemented HA (**Figure 4**). In NS groups under HA conditions, OHA significantly increased ICAM1 mRNA expression by 9.5-fold change (*p<*0.001), while HHA slightly increased ICAM1 mRNA expression by 2.5-fold change (*p=*0.011). In terms of CHA, it did not cause an upregulation compared to control, the mRNA expression was lower than that induced by HHA (*p*<0.006).

In BS group, all HA groups decreased by trend ICAM1 mRNA (not statistically significant).

#### 3.2.3 Oxidative stress in MONO-MAC-6 cells

The total ROS level was quantified to investigate whether the biofilm burden caused oxidative stress in MONO-MAC-6 cells, and to evaluate the effect of HA as potential antioxidant under these conditions. Medium without or containing 4 mg/mL of HAs was applied to the cells and those were exposed to the biofilm or not for 1 h, before subsequently the ROS level was measured.

**Figure 5:**
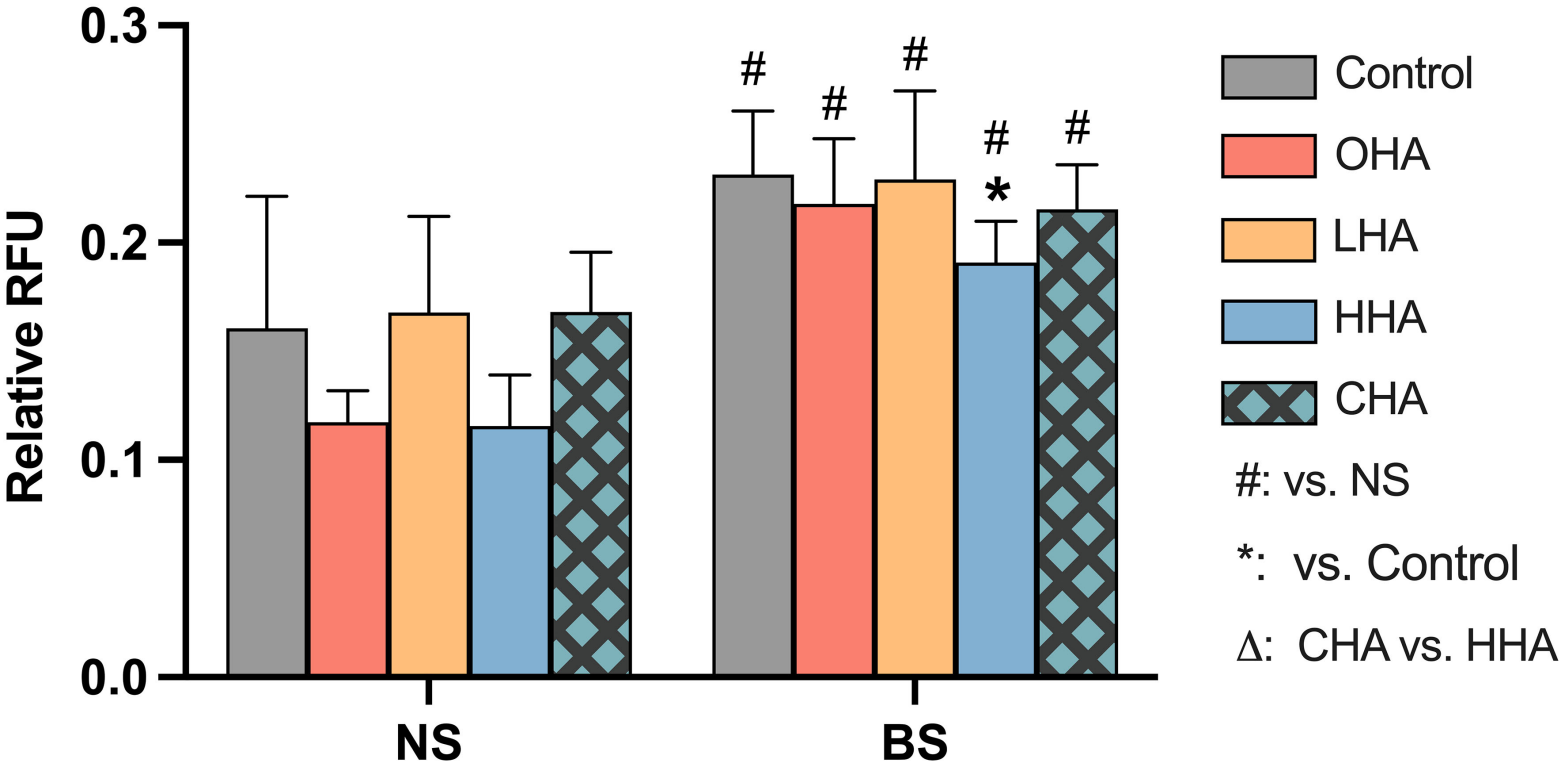
Effect of 4 mg/ml hyaluronic acid (HA) (OHA: 6 kDa, non-cross-linked; LHA: 400 kDa, non-cross-linked; HHA: 1000 kDa, non-cross-linked; CHA: 1000 kDa, cross-linked) on the total amount of reactive oxygen species (ROS) in a monocytic cell line (MONO-MAC-6 cells) with or without periodontal biofilm after 2 h of stimulation. NS, non-biofilm-stimulated; BS, biofilm-stimulated. Mean ± SD, */** *p*<0.05/*p*<0.01 vs. control, △ *p*<0.05 vs. HHA group and # *p*<0.05 vs. the corresponding group in NS cells.

According to **Figure 5**, biofilm stimulation significantly increased ROS levels compared to NS groups in the corresponding medium condition [*p<*0.01 in all groups (with and without HAs)]. In NS groups, the addition of HA to MONO-MAC-6 cells did not significantly affect the ROS levels (*p*>0.05 in all groups vs. control). In the BS groups, only the HHA treatment showed a statistically significant reduction in ROS vs. BS control (*p=*0.039).

### 3.3 Immune interaction between PDLF and biofilm stimulation

The inflammatory response of PDLF with and without biofilm lysate stimulation was investigated. As before several aspects were examined, including expression of inflammatory chemokine, HA receptors, and oxidative stress.

As before the cell viability was tested under the experimental conditions, it was always more than 75%.

#### 3.3.1 Expression of IL-8 in PDLF

Protein and mRNA expressions of IL-8 were evaluated in PDLF. Cells were cultured in either regular medium or HAs-medium (4 mg/ml) and were exposed in part to biofilm supernatants. The release of IL-8 protein was measured after 2 h and 4 h, while the expression of IL-8 mRNA was measured after 2 h.

**Figure 6:**
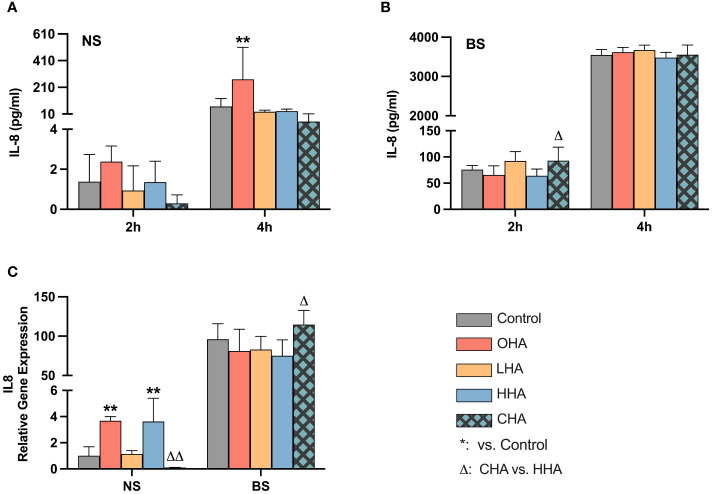
Effect of 4 mg/ml hyaluronic acid (HA) with different molecular weights (OHA: 6 kDa, non-cross-linked; LHA: 400 kDa, non-cross-linked; HHA: 1000 kDa, non-cross-linked; CHA: 1000 kDa, cross-linked) on protein **(A, B)** and mRNA **(C)** expression of interleukin-8 (IL-8, IL8) in periodontal ligament fibroblasts (PDLF) with **(B, C)** or without **(A, C)** periodontal biofilm after 2 h and 4 h (only protein levels) of stimulation. NS, non-biofilm-stimulated; BS, biofilm-stimulated. Mean ± SD, */** *p*<0.05/*p*<0.01 vs. control, △ *p*<0.05 vs. HHA group.

The release of IL-8 protein in PDLF increased over time in the corresponding medium in both NS and BS groups (**Figures 6A, B**). In the absence of HA, biofilm significantly increased IL-8 protein and mRNA expression vs. NS group (each *p*<0.001, **Figures 6B, C**).

In the NS groups, OHA increased IL-8 protein expression at 4 h (*p<*0.001). At the mRNA level, OHA and HHA increased IL8 mRNA expression with 3.7-fold and 3.6-fold change, respectively (both *p<*0.001). In contrast to HHA, CHA caused by trend a downregulation of IL8 mRNA expression to 0.1-fold of control (*p*=0.402) which is 0.03-fold of HHA (*p<*0.001).

In the BS group, there were no significant differences observed between HA groups and controls at the protein level after 2 h and 4 h (**Figure 6B**) and at the mRNA level after 2 h (**Figure 6C**). CHA slightly increased IL-8 protein level compared to HHA at 2 h (*p*=0.002). Also, the IL8 mRNA expression was increased by CHA by 1.5-fold compared to HHA (*p=*0.023).

#### 3.3.2 Expression of HA receptors in PDLF

As in MONO-MAC-6-cells, HA receptor genes (CD44, RHAMM, TLR2, TLR4 and ICAM1) were also examined in PDLF after 2 h exposure to biofilm supernatants in medium without or with HAs (4 mg/ml).

**Figure 7:**
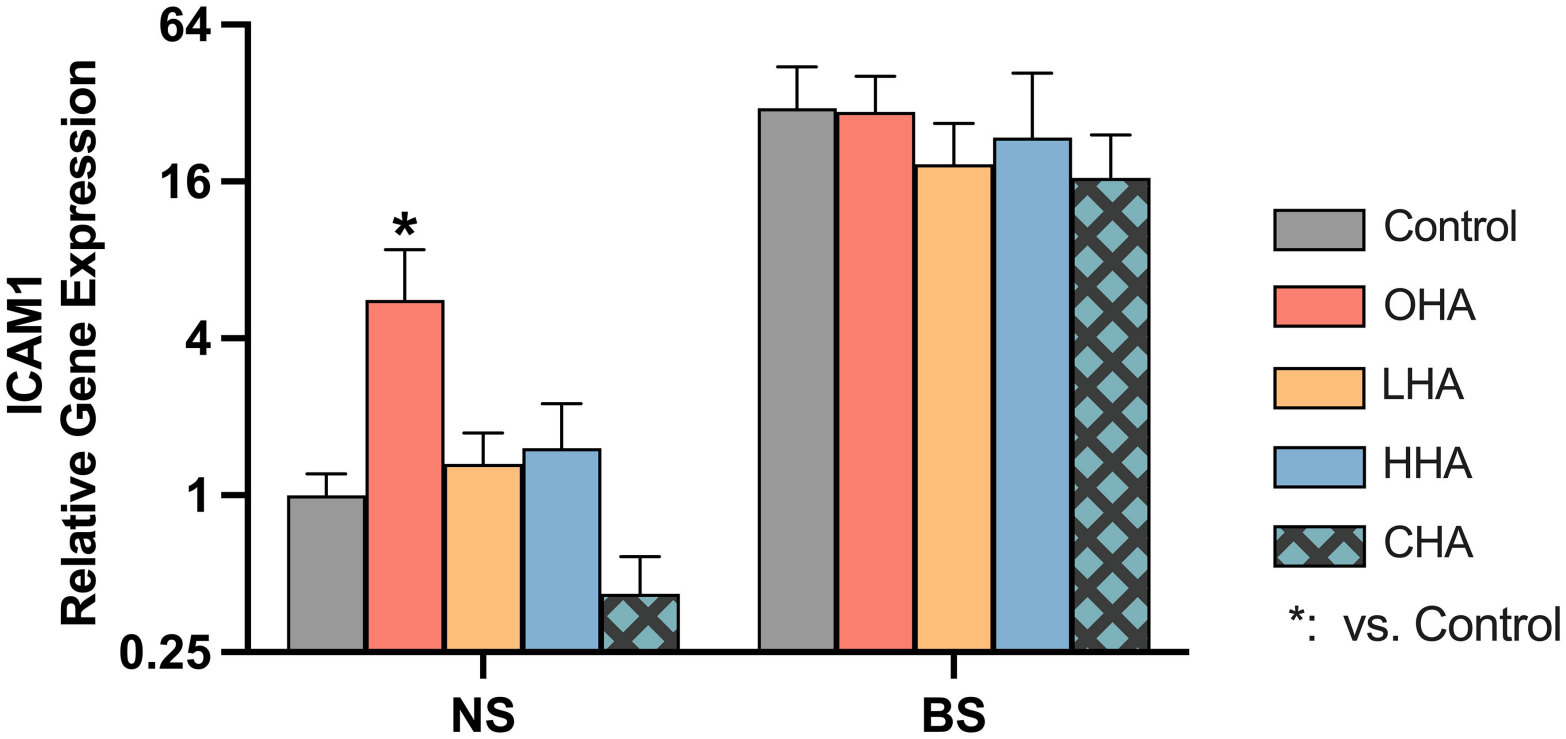
Effect of 4 mg/ml hyaluronic acid (HA) (OHA: 6 kDa, non-cross-linked; LHA: 400 kDa, non-cross-linked; HHA: 1000 kDa, non-cross-linked; CHA: 1000 kDa, cross-linked) on the mRNA expression levels of a HA receptor gene – ICAM1 in periodontal ligament fibroblasts (PDLF). with or without biofilm after 2 h stimulation. NS, non-biofilm-stimulated; BS, biofilm-stimulated, Mean ± SD, * *p*<0.05 vs. control.

As before for the MONO-MAC-6-cells, there were only differences between the groups found for the ICAM mRNA expression. Following only the ICAM results are presented (**Figure 7**).

The biofilm supernatant increased ICAM1 mRNA expression in PDLF, causing a 30.6-fold upregulation without HA. In the NS group, OHA increased ICAM1 mRNA expression by 5.6-fold change (*p*<0.001). In the BS group, LHA, HHA, CHA seemed to decrease in ICAM1 mRNA expression, although it was not statistically significant.

#### 3.3.3 Oxidative stress in PDLF

**Figure 8:**
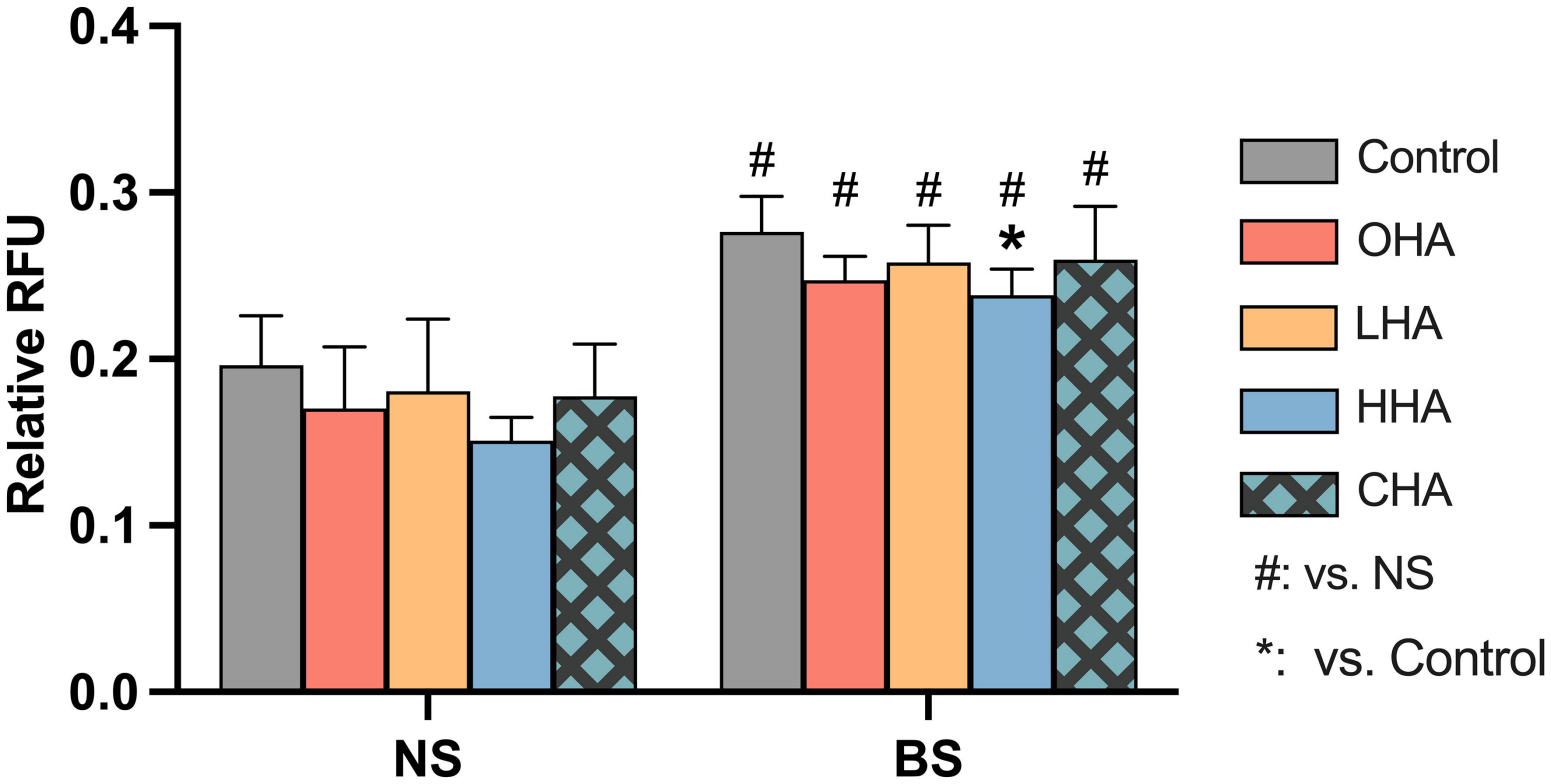
Effect of 4 mg/ml hyaluronic acid (HA) (OHA: 6 kDa, non-cross-linked; LHA: 400 kDa, non-cross-linked; HHA: 1000 kDa, non-cross-linked; CHA: 1000 kDa, cross-linked) on the total amount of reactive oxygen species (ROS) in periodontal ligament fibroblasts (PDLF) with or without periodontal biofilm after 1 h of stimulation. NS, non-stimulated; BS, biofilm-stimulated. Mean ± SD, * *p*<0.05 vs. control and # *p*<0.05 vs. the corresponding group in NS cells.

The total level of ROS was measured in PDLF after 1 h exposure to HA medium (4 mg/ml) and biofilm stimulation (**Figure 8**).

Biofilm stimulation significantly increased ROS generation in PDLF (*p<*0.001). In the NS group, there was no statistical significance observed among all groups. In the BS group, the addition of HA resulted in statistical significance only in the HHA group (*p*=0.046) in reducing the high level of ROS induced upon biofilm stimulation.

## 4 Discussion

The objective of this study was to investigate the potential effects of different molecular weights (MW) of HA, as well as two different formulations (cross-linked vs. non-cross-linked), on the interaction between periodontal biofilm and immune cells. This study is the first to report the potential anti-biofilm, anti-oxidative, and anti-inflammatory properties of various HAs when directly challenged by a 12-species periodontal biofilm. The results underscore that HA influenced the periodontal biofilm itself and modulated the immune response of oral cells, with the effect depending on the MW and chemical modification (cross-linked or non-cross-linked).

Our primary objective was to investigate the effect of different HAs on the 12-species periodontal biofilms. None of the tested HAs (up to 10 mg/ml) clearly inhibited the growth of the included 11 oral species. Data on MIC values of HA alone on planktonic bacteria are scarce. However, higher concentrations of 40 mg/ml were found to be growth inhibitory against β-hemolytic streptococci, *Staphylococcus aureus*, *S. epidermidis* (Carlson et al., 2004). However, our results differ from those of other studies that determined an MIC of 4 mg/ml against *P. gingivalis* (Alharbi and Alshehri, 2022).

Conversely, the two types of high-MW HA inhibited the formation of the tested 12-species periodontal biofilm in a concentration-dependent manner, with CHA showing the strongest effect in the present study. This anti-biofilm formation ability of HAs aligns with the findings from other studies.

For example, HA inhibited single biofilm formation, demonstrating a more sensitive effect on the biofilm produced by *Staphylococcus aureus* than *Haemophilus influenzae* and *Moraxella catarrhalis* (Drago et al., 2014). In general, a hydrophilic and negatively charged surface prevents bacteria adhesion (Delaviz et al., 2015). In this study, the negatively charged HA may form a hydrophilic layer on the mucin-BSA-coated surface, which may prevent planktonic bacteria from attaching to the proteinaceous surface, thereby inhibiting the initial stage of biofilm formation (Hannig and Hannig, 2009; Fallacara et al., 2018). The extent of the formed hydrophilic layer is correlated with the MW size (Fallacara et al., 2018) which may explain that only the high-MW HAs (HHA and CHA) act inhibitory. Regarding the stronger effect of CHA, its cross-linked structure provides a network of layers that slows down diffusion and serves as a more stable barrier (De Boulle et al., 2013).

Another essential aspect is the impact of HA on destruction of a pre-existing biofilm. The analysis revealed that both HHA and CHA reduced the biomass. This effect could potentially be attributed to their ability to dissolve the biofilm matrix, as there were no changes observed in the CFU counts or reductions in the metabolic activity of the biofilm microorganisms. Similarly, Champion et al. demonstrated that high-MW HA affected the biomass but not the bacterial counts of already-formed *P. aeruginosa* biofilm (Champion et al., 2022). Investigating the potential destruction of biofilm matrix might be of interest in upcoming studies.

The *in-vitro* findings on the anti-biofilm effect of HA might support clinical data on oral bacteria, where applying high MW HA gel in the peri-implant sulcus for 45 days reduced the relative abundance of peri-implantitis-related microorganisms, particularly *Prevotella and Campylobacter* (Soriano-Lerma et al., 2020).

Periodontal disease is not solely caused by biofilm itself, it results also from the host immune response to the microbes (Cekici et al., 2014). In the dynamic development of periodontitis, macrophages play an essential role in immune regulation and phagocytosis by differentiating to different phenotypes (Sun et al., 2021). The M1 phenotype promotes killing of bacteria and increases inflammation by producing high levels of pro-inflammatory cytokines like IL-1β and TNF-α. In contrast, the M2 phenotype induces tissue regeneration by releasing anti-inflammatory cytokines like IL-10 and TGF-β (Orekhov et al., 2019). The M1/M2 ratio was found to be increased in periodontitis (Yu et al., 2016). This may be supported by the present *in-vitro* study with highly elevated levels of IL-1β and decreased levels of IL-10 in MONO-MAC-6 cells after biofilm stimulation.

The effects of HA on macrophages are closely related to its MW. As investigated with murine macrophages, regardless of the initial polarization state of macrophages, macrophages underwent phenotypic alterations based on MW of HA, which was a pro-inflammatory response for lower MW HA and oligos of HA (no more than 5 kDa) and a pro-resolving response for higher MW HA (3000 kDa), while the response to intermediate-MW HA (60 kDa – 800 kDa) was difficult to ascertain (Rayahin et al., 2015). In a similar study, high-MW HA (1500 kDa) caused a concentration-dependent reduction in IL-1β in murine macrophages stimulated with LPS, while low-MW HA (100 kDa and 500 kDa) caused an increase in IL-1β. But at both 500 kDa and 1500 kDa MW, HA increased IL-10 in LPS-stimulated macrophages (Lee et al., 2021). In the present study, we found that without biofilm stimulation, OHA induced both IL-1β and IL-10 in MONO-MAC-6 cells, while with biofilm stimulation, high-MW HA (HHA and CHA) decreased IL-1β and increased IL-10. Together with our findings, it suggests that OHA promotes inflammation whereas high-MW HA decreases inflammation induced by bacterial stimulus.

The contrasting response to inflammation in MONO-MAC-6 cells to the different molecular weights (MWs) of HA may correspond with varying affinities to the receptors, which subsequently affect the downstream signaling pathways (Yang et al., 2012). CD44, TLR2/4, RHAMM and LYE-1, ICAM-1 have been reported as the receptors of HA (Vasvani et al., 2020). However, except for ICAM-1, we did not find any difference in the mRNA expression of these receptors in MONO-MAC-6 cells with or without biofilm stimulation in this study. Both HHA and in particular, OHA increased ICAM1 mRNA expression, which suggested that HA fragments and high-MW HA had a higher affinity to ICAM-1 in MONO-MAC-6 cells. The role of ICAM-1 in HA signaling is underlined by an *in-vitro* study with LPS-stimulated human U937 macrophages [51]. The decreasing effect of high-MW HA on pro-inflammatory cytokines as IL-1β, IL-6 and TNF-α was mitigated when an anti-ICAM-1 antibody was applied prior to HA incubation (Yasuda, 2007). The HA binding to ICAM-1 down-regulated p65 NF-kB phosphorylation without affecting MAPK pathways (Yasuda, 2007).

Periodontal ligament fibroblasts (PDLF) are also a crucial and prominent cell type in periodontal homeostasis and regeneration due to their ability to produce multiple cytokines in response to bacterial insults, including the proinflammatory chemokine IL-8 (El-Awady et al., 2010). IL-8 is among the most abundant chemokines in periodontitis which functions as attracting PMNs to infectious area and affecting bone metabolism (Sahingur and Yeudall, 2015). High IL-8 levels were found *in vivo* in periodontitis patients (Chen et al., 2015) and *in vitro* in bacteria-stimulated periodontal fibroblasts (Makkar et al., 2022). In the present study, biofilm stimulation enormously increased the level of IL-8 in PDLF as well. The PDLF cells respond differently to HA depending on its MW. In this study, OHA promoted the production of IL-8 in resting PDLF time-dependently. Nakatani et al. revealed that HA oligomers increased matrix metalloproteinase-1 in PDLF via p38MAPK signaling pathway (Nakatani et al., 2009). These and our findings suggest that OHA may promote periodontal tissue degradation under the pathologic conditions. High-MW HA (1300 kDa) was reported to downregulate IL-8 in *P. gingivalis*-stimulated gingival fibroblasts (Chen et al., 2019). This study found a downregulation by HHA (1000 kDa) with or without biofilm stimulation, although the effect was not statistically significant.

As with the MONO-MAC-6 cells, an influence of HA on its receptor expression was only found for ICAM-1. Most expression was stimulated by the periodontal biofilm. The increased expression of ICAM-1 in periodontal fibroblasts was shown for *P. gingivalis*, a member of our multi-species biofilm (Liu et al., 2014). In the presence of biofilm, not any HA could significantly influence the expression underlying the overwhelming role of bacteria. Our data indicated that in the absence of bacterial stimuli, OHA increased the expression of ICAM-1 and thus may support inflammation in the periodontium. In periodontal junctional epithelium, a gradient expression of ICAM-1 together with the high releasing of IL-8 are thought to be an important mechanism for guiding PMNs to infected areas, for example: the bottom of sulcus, where they are directly challenged by bacteria and their components (Tonetti et al., 1998).

ROS generation plays a critical role in numerous diseases, including periodontitis. ROS kills bacteria in high amounts, but when overactivated, it becomes cytotoxic to host cells, leading to tissue destruction (Sies et al., 2022). Several studies have shown a direct correlation between elevated oxidative stress and periodontitis (Sczepanik et al., 2020). Our data showed that periodontal biofilm induced oxidative stress in host immune cells as evidenced by the higher ROS levels in both MONO-MAC-6 and PDLF. Addition of antioxidants to conventional approaches may be an option for the prevention and treatment of periodontitis (Sczepanik et al., 2020). In the present study, HHA slightly retarded the high ROS generated by biofilm stimulation. High-MW HA was shown to have antioxidant capacity by scavenging excessive ROS (Šoltés et al., 2006). Clinically, adjunctive application of high-MW HA led to a higher increase in antioxidant markers in saliva when compared to non-surgical periodontal therapy without adjunct (Olszewska-Czyz et al., 2022).

It is worth to note that in the context of two immune cell types, the two high-MW HA affect differently on inflammatory response including cytokine expression and ROS generation. Without biofilm stimulation, CHA reduced IL-10 levels in MONO-MAC-6 cells and IL-8 levels in PDLF indicating a potential inhibitory effect of CHA on immune cells. In biofilm-stimulated MONO-MAC-6 cells, CHA reduced IL-1β levels less than HHA but increased IL-10 levels more. According to the manufacturer’s information, CHA contains the crosslinker 1,4-butanediol diglycidyl ether (BDDE). BBDE is one of the most used cross-linker in commercial HA products (De Boulle et al., 2013). It might be responsible for the higher level of IL-1beta in CHA compared to HHA. BBDE has been shown to induce higher levels of pro-inflammatory cytokines, such as TNF-α and IL-1β than other crosslinkers in human keratinocyte cell line and human dermal fibroblast cell line (Jeong et al., 2021). Higher levels of ROS were observed in CHA group than in HHA group. The crosslinked structure appeared to be a feasible way to protect the long HHA chains from rapid degradation by free ROS (De Boulle et al., 2013).

In summary, within the tested 12-species periodontal biofilm, both high-MW HAs exhibited anti-biofilm capacity against early-stage and mature biofilms, with CHA demonstrating the most significant effect. Concerning periodontal immune cells and their interaction with biofilm, OHA might initiate a pro-inflammatory response in both MONO-MAC-6 cells and PDLF, whereas CHA inhibited it. When challenged with biofilm, HHA and CHA reduced pro-inflammatory IL-1β, while CHA increased the level of the anti-inflammatory IL-10. Expression of ICAM-1 is involved in the interaction between HA, biofilm, and MONO-MAC-6 as well as PDLF cells.

Nevertheless, this study has limitations. While this study is the first to demonstrate the anti-biofilm effects of HHA and CHA on the 12-species periodontal biofilm with defined strains, we acknowledge that we did not investigate the complexity as it occurs *in vivo*, nor the effect on the virulent factors of periodontal pathogens. Furthermore, the current study did not examine in detail how HA is involved in the M1/M2 switch of macrophages or in the potential signaling pathways. Despite the promising effect of HA on biofilm and immune cells, the effect of the biofilm and immune cells on HA was not examined. Future studies on this topic are of interest.

Overall, this study explored the effects of different types of HA, including variations in molecular weight (MW) and cross-link formulations, on periodontal biofilm, immune cells, and their interactions. The findings suggest that utilizing different types of HA at specific times and conditions during periodontal treatment may enhance the benefits of HA and improve clinical outcomes. However, further evidence is needed, and additional studies should focus on this aspect.

## Data availability statement

The raw data supporting the conclusions of this article will be made available by the authors, without undue reservation.

## Ethics statement

Ethical approval was not required for the studies involving humans because According to the criteria of the Cantonal Ethical Committee (KEK), there is no need for additional approval if the biomaterials are classified as “irreversibly anonymized”. The studies were conducted in accordance with the local legislation and institutional requirements. The participants provided their written informed consent to participate in this study.

## Author contributions

XZ: Conceptualization, Data curation, Formal analysis, Funding acquisition, Investigation, Methodology, Writing – original draft, Writing – review & editing. AS: Resources, Supervision, Writing – review & editing. SE: Conceptualization, Data curation, Investigation, Methodology, Project administration, Supervision, Writing – review & editing.
